# Assessment of Adaptive Behavior in People with Autism Spectrum Disorders through the ICAP

**DOI:** 10.3390/bs12090333

**Published:** 2022-09-15

**Authors:** Luisa Losada-Puente, Manoel Baña

**Affiliations:** 1 Department of Methods of Research and Diagnosis in Education, Faculty of Educational Sciences, University of A Coruña, 15071 A Coruña, Spain; 2 Department of Developmental and Educational Psychology, Faculty of Teachers Training, University of Santiago de Compostela, 15782 Santiago de Compostela, Spain

**Keywords:** adaptive behavior, autism spectrum disorder, assessment, measurement properties

## Abstract

Evaluating adaptive behavior in people with Autism Spectrum Disorder (ASD) requires attending to a set of cognitive processes associated with social interaction skills and functional communication that are altered. This paper presents the analysis of an instrument to assess and diagnose adaptive behavior in people with Autism Spectrum Disorder (ASD), given the need for rigorous, standardized, and statistically reliable tools to address this dimension, incorporated into the diagnosis since 1992. The Inventory for Service Planning and Individual Programming (ICAP) was applied to *n* = 209 children with ASD. Its psychometric properties were studied to provide statistical criteria for its usefulness in assessing adaptive behavior. Results highlighted variations in its original structure, reducing the number of items from 77 to 60 by eliminating those with little discriminative power, and of dimensions from four to three given their greater congruence with the results of the exploratory analysis: daily life skills (α = 0.892–0.935), communication and linguistic skills (α = 0.860–0.931), and motor skills (α = 0.828–0.857). This again raises questions about the use of instruments similar in their dimensions, and about the interaction between variables and items, a frequent issue in the field of mind, social, and health sciences.

## 1. Introduction

Adaptive behavior refers to the set of daily activities, including behaviors essential for social communication and interaction [1,2,3]. This concept has gained relevance since its inclusion in the definition of Intellectual Disability proposed by Luckasson and collaborators [4], causing great debate and controversy around its meaning and use. Today, it is recognized as one of the parameters or criteria that are introduced within an inclusive definition of disability [5], representing a useful concept to explain many of the behaviors and actions of people with Autism Spectrum Disorder (ASD) [6,7] or even “a key phenotypic construct in autism spectrum disorder” [8]. 

The alterations in the development of these basic life and social interaction skills occur frequently in children with ASD. These alterations affect their learning processes, functioning, and school performance and make them less effective than children with neurotypical development given their low levels of motivation and flexibility, their difficulties in problem-solving or their low persistence on a task [9,10,11]. In general, this has an impact on their development and on a full and quality coexistence [12,13,14]. Children with ASD also have a more negative approach to learning and more problems in their relationship with their peers in the classroom [7,15]. Those social disturbances increase the risk of bigger academic maladjustment which is manifested in a negative attitude toward school activities, teachers, and learning materials [10,16,17].

Evaluating adaptive behavior involves checking the practical and real performance of conceptual, social, and practical adaptative skills and specific environments in which the person lives, cohabitates, mingles, gets involved, makes decisions… This enables us to analyze the abilities displayed and the difficulties encountered by the individuals in their environments and determine the support needed for their independent and autonomous life [5,18,19,20]. The introduction of this concept in the classification of neurodevelopmental disorders has marked the beginning of great work by researchers and professionals who have set out to describe, conceptualize and objectify the components and aspects of adaptive behaviors [3]), recognizing the importance of its evaluation as a specific criterion for diagnosing intellectual and developmental disabilities [5,14,21]. Specifically, the DSM-5 (American Psychiatric Association [22]) defines Intellectual Developmental Disorder within Neurodevelopmental Disorders, as a group of conditions with onset in the developmental period that includes limitations in intellectual functioning, as well as adaptive behavior in the conceptual, social, and practical domains.

The concept of adaptive behavior began to enter the academic and clinical debate in the 1970s. However, the breakthrough came with the publication of the 10th edition of the American Association on Mental Retardation (AAMR) Manual [23], in which it is introduced as an added dimension to the previous model [4]. In this manual, adaptive behavior is used, eliminating adaptive skills, although it is expressed in terms of conceptual, practical, and social adaptive skills related to Greenspan’s research on personal, social, and intellectual competence [24,25]. Since then, adaptive behavior has been defined as a complex construct that includes the combination of three elements: academic (conceptual) performance, functional (practical) skills, and social (societal) skills [2], being linked with other concepts such as intelligence [3,15,26], quality of life [7,12,27] and self-determination [28,29]. Anyone who analyzes a broad enough adaptive behavior tool will realize that self-determination in one’s own life can hardly be performed without starting from the solid foundation provided by the set of skills that a citizen needs to cope with a minimum of success with the behavioral challenges of every day [29].

Moreover, with the publication of the first edition of the American Association on Intellectual and Developmental Disabilities (AAIDD) Manual [27], adaptive behavior was introduced as one of the five dimensions of the intellectual disability model. Thus, the AAMR [30] reinforces its idea of including the intellectual disability model of intellectual disability in that of the World Health Organization’s (WHO) model of human development, expressed in the International Classification of Functioning, Disability, and Health. Quality of life is defined as a multidimensional concept, spanning several domains that reflect positive values, and life experiences that, at the same time, participate in the desired states that are linked to personal well-being [31]. These domains, expressed by different models of quality of life [26,32,33,34] are related to adaptive behavior. Therefore, the 10th edition of the AAMR Handbook [23] incorporates a new framework for assessing intellectual disability and adaptive behavior, outlining its different functions: diagnosing, classifying, and planning supports.

Therefore, the evaluation of adaptive behavior and skills has evolved substantially over the last decades. At present, the quality of most of the instruments used, both in their evaluation and in the care processes, has increased and improved [3], although there is still a low number of instruments that take as base the structure proposed by the AAIDD and that have sufficient evidence of reliability and validity [21,35]. Despite the attempts carried out, it is difficult to build an instrument that would encompass the needs and demands that can arise from the diversity of actions, environments, and contexts of life, and especially in people with ASD whose adaptive behavior is considered especially *heterogeneous and variable—*even within an individual—since as Farmer and collaborators [8] pointed out “while adaptive behavior impairment has a generally predictable relationship with cognitive ability in samples of individuals with intellectual disability, the relationship appears to be more complicated in individuals with ASD” (p. 2). Although the bulk of knowledge about adaptive behavior in individuals with ASD comes from cross-sectional studies [9], recent data show that some phenotypic characteristics may be less stable over time [8,36]. Specifically, the longitudinal study of Farmer et al. [8] showed that the degree of ability and impairment associated with ASD and its types of manifestations across the lifespan may lead to differences in independent living behaviors in children with ASD. Other studies have shown that executive function skills alone are predictors of significant variance in autistic traits, even controlling for variables such as age or intellectual ability, as well as adaptive behavior in young people followed for 12 years [36] or assessed between two and five times, with a six-month window [37].

There is still no resource available for people with ASD and other developmental disabilities that allows evaluating normatively and with criteria, homogeneously and satisfactorily when it comes to evaluating both for diagnostic purposes and to proceed with personalized development plans [7]. To solve this problem, professionals resort to testing sets and instruments that are used in diverse ways, depending on the purpose pursued in specific situations. It often happens that fitting those instruments is not easy given their provenance and origin, as well as the purpose for which they were created, their conceptual and technical coherence [14,38,39].

Indeed, the growing importance of adaptive behavior because of its introduction in the evaluation of intellectual disability has not meant considerable progress, still being at the stage of development of instruments and resources for diagnosis. Tassé and collaborators [20] referred to a paper by Schalock in 1999, who identified 200 tests of adaptive behavior, of which Tassé and colleagues [19] found only four valid to be applied in an English setting to make a diagnosis of significant difficulties. These were: Adaptive Behaviour Assessment System, second edition (ABAS-2), developed by Harrison & Oakland in 2003; Adaptive Behaviour Scale-School-based, second edition (ABS-S: 2) by Lambert, Nihira, & Leland in 1993; the Scales of Independent Behaviour-Revised (SIB-R) by Bruininks, Woodcock, Weatherman, & Hill in 1996, and the Vineland Adaptive Behaviour Scale, second edition (VABS-2) by Sparrow, Cicchetti, & Balla in 2005. The selection of these instruments was justified by their potential to measure adaptive behavior in terms of specific adaptive skills reflecting a multidimensional conceptual and measurement model. However, all of them were designed to assess the full range of individual differences in adaptive behavior without attending to the particularities of individuals with developmental disabilities and, particularly, with ASD.

Recent studies continue to present difficulties in unifying the behavioral indicators that include conceptual, social, and practical skills [2,3,21], so the study and analysis of those tests must be the object of continuous research, adapting them to its purposes and seeking relevant resources for evaluation and care of people with ASD and their families. Therefore, the objective of this study is to validate the most significant adaptive behavior assessment instrument of recent years, both in research and professionally for the development of person-centered plans in Spain: Inventory for Client and Agency Planning (the abbreviations are used in the original language: ICAP) [40].

## 2. Materials and Methods

Given that the purpose of this study was to test the psychometric properties of an instrument specifically designed to assess a person’s adaptive behavior, the instrument used for this study, the Inventory for Service Planning, and Individual Programming (ICAP) was applied and tested in a Spanish sample of 209 individuals (Male: *n =* 116, 55.5%; Female: *n =* 93, 44.5%) with ASD, between 2–23 years of age (M = 10.20; DT = 4.84). Specifically, there were 14 children between 2–4 years (6.7%), 61 between 5–7 years (29.2%), 44 between 8–10 years (21.1%), 35 between 11–13 years (16.7%), 21 between 14–16 years (10%), 28 between 17–19 years (13.4%) and 6 between 20–23 years (2.9%). All participants were institutionalized at the time of application of the instruments, in ordinary (*n* = 55, 26.3%) or specific (154, 73.7%) schools. The youngest children (2–3 years old: *n* = 4, 1.94%) were in regular kindergartens.

All participants took part in the study voluntarily, having been previously informed about the characteristics and objectives of the study, and about the confidentiality of the provided data. It was not necessary to ask for the family’s consent, as the families were themselves recruited by snowball sampling initiated through contacts with specialized ASD institutions and completed the instrument. The ICAP was the only instrument used for the study. The criterion for participant selection is that of access to the sample through direct contact with ordinary and special education centers and the willingness of the families to be part of the study

In general, ASD was the only pathology present (*n* = 168, 80.4%), however, there were informed by families about cases of secondary pathologies of blindness (*n* = 3, 1.4%), brain injury (*n* = 8, 3.8%), cerebral palsy (n = 2, 1%), intellectual disability (*n* = 14, 6.7%) or physical disability (*n* = 14, 6.7%). There were more boys (*n* = 116, 55.5%) than girls (*n* = 93, 44.5%) in the sample, and more students schooled in special education centers (*n* = 154, 73.7%) than in ordinary education centers (*n* = 55, 26.3%). Most of the participants used verbal communication as a means of expression (*n* = 175, 83.7%) and, to a lesser extent, non-verbal communication (*n* = 34, 16.3%). The need for support (*n* = 20, 9.6%), notable support (*n* = 11, 5.3%) or significant support (*n* = 16, 7.7%) is lower than the absence of support (*n* = 162, 77.5%).

As for the main characteristics of the instrument, it can be said that the ICAP is an inventory created by Bruininks, Hill, Woodcock, and Wearherman, translated and adapted into the Spanish language by Montero (from Deusto University). It is composed of a systematic record of relevant data on the person attending a service and of two normative measurement instruments, one for adaptive behavior and the other for behavior problems. The ICAP aims to assess the functionality, adaptive behavior, behavioral problems, and services of people of all ages with disabilities, although it can be utilized with other types of populations that may differ from the previous category.

It uses a self-report type format to be answered in a variable time (approx. 30–45 min) by any guardian, caregiver, teacher, or professional who has known the person for, at least, three months (in this study, it was the family who completed the Spanish version of the questionnaire, receiving support from researchers, when needed). It is composed of 77 items that include:

1. A record of the person’s diagnosis or diagnoses, personal data, and functional difficulties (mobility, vision, hearing, and health status).
2. An adaptive behavior test that measures the level of the person in relation to basic skills to function independently in their environment and is structured in four scales: linguistic and communicative skills (LCS-expressive and receptive language), daily life skills (DLS-independence in the realization of immediate personal needs), community life skills (CLS-independence in activities such as using public transport, handling money, using the watch…) and motor skills (MS-fine and gross). Those items must be answered on a 4-point Likert scale (0-Never/rarely does it; 3-Does it very well). For each scale, plus a general one that encompasses them, normative scores are established: age, percentiles, and typical scores, among others, as well as an Instructional Implications Profile in which two ages are obtained, between which the content of the specific programs for the evaluated person could be placed (adjusting the level of difficulty).
3. A test of behavior problems, focused on eight areas from which four normative indices of behavior problems are extracted: Internal, Asocial, External, and General. For its evaluation, the degree of severity and the frequency of these behaviors are used. The response that these behaviors usually received from significant people in the individual’s environment are also recorded.

The ICAP was typified and regarding its reliability in the Spanish context, the research carried out, especially in samples of people with disabilities, showed that it has adequate internal consistency (*α* = 0.8) and the estimates made by independent evaluators are consistent [40,41]. Later studies offered additional evidence of its reliability with people with (*α* = 0.88–0.98) and without disabilities (*α* = 0.86–0.98). Recently, its reliability has also been studied in Chile [35,42] with excellent results. Likewise, their divergent validity was confirmed by Vera-Bachmann and colleagues [35] for the Chilean population, by comparing the four scales of the ICAP and the Parenting Styles Scale through Pearson’s r coefficient.

The validation of the instrument was carried out in this study in several phases and activities, all conducted with the support of IBM SPSS 27.0 (Statistical Package for Social Sciences) (IBM, Version is 27.0.1., Armonk, NY, USA). Note that the Spanish version was used, which was subsequently translated into English based on the original version for publication purposes.

Before starting the analysis, the data are prepared for statistical treatment by the imputation of missing values. As these are Missing at Random (MAR) values, which are predictable from the observed components of other variables in the dataset [43], the multiple imputation method was used. This method allows multiple linear regression estimates to be computed by augmenting the estimates with random components where, for each predicted value, a randomly selected residual from a complete case, a random normal deviation, or a random deviation from the Student-T distribution can be added. This procedure was performed through the SPSS program automatically. After that, a correlational analysis of the items and of the reliability was conducted. This involved the elimination of items whose properties did not meet the minimum requirements. Once this procedure was completed, an exploratory factor analysis (EFA) was used to identify the main dimensions of the instrument. An exploratory rather than a confirmatory factor analysis was chosen because, despite having a basic theoretical framework that indicated the presence of four dimensions of the questionnaire applicable to the population, everything suggested that it would be necessary to explore the behavior of the instrument in a specific population, such as that of people with ASD.

## 3. Results

### 3.1. Correlational Analysis of the Items and Reliability of the Scale

First, the correlation between each item on the scale and the total score is analyzed, checking the discriminatory power of each item in each test, eliminating the contribution of the latter to the item using the Corrected Homogeneity Index (cHI). The Discrimination Index (DI) is also studied. Finally, the reliability is checked through Cronbach’s Alpha, studying the increase of this index if any element of each scale is eliminated.

Low scores were obtained in items of the linguistic and communicative skills dimension (LCS), referring to advanced skills and more typical of adulthood, such as locating information or hiring repair services (LCS18, LCS19). Also, in advanced skills in daily life skills (DLS20, DLS21) and life in the community (CLS17, CLS18, CLS19). The highest values have been obtained in basic motor skills (MS4, MS5). The CHI has been, in general, higher than 1.5 points, with several exceptions. In the basic motor skills dimension, values lower than expected were obtained in the items MS3 (“Sits alone, keeping the head and back straight and firm for 30 s”), MS4 (“Stands, at least for 5 s, leaning on furniture or other objects”) and MS7 (“Stands without help and shifts, at least, about two meters”). Likewise, the items contained in the dimension of linguistic and communicative skills LCS1 and LCS2 present values lower than 1.5 points and close to the value of 0. Given the negative nature of those two items, their exclusion is recommended [44].

The discrimination index [DI = (AS − AI)/(N/2)] of most of the items offered values between DI = 0.10–0.95, which reflected a high discriminatory power. Some items presented a moderate discrimination power, with DI values < 0.10 (MS3, MS5, DLS2) and, in four cases, lower than DI = 0.05 (MS4, MS7, LCS1, LCS2), making it necessary to consider their elimination. In the case of items MS3 (“Sits alone, keeping the head and back straight and steady for 30 s”), MS4 (“Stands up for at least 5 s, leaning on furniture or other objects ”), MS5 (“Stands up by himself/herself”) and MS7 (“Stands without help and walks at least 1–2 m”), their similar content and the high correlations between them (*r* < 0.80), led to the elimination of those two that obtained a lower DI (MS4 ID = 0.03; MS7 ID = 0.06), keeping the item MS5 (DI = 0.08) and the item MS3 (DI = 0.10).

Item DLS2 (“Takes and eats food such as cookies or chips”) was eliminated by presenting a DI < 0.10 and presenting limitations at the item construction level since it contemplates two concepts in the same item (“take” and “eat”), as well as information related to that of the previous item (DVP1). Finally, the items DLC1 (“Makes sounds or gestures to attract attention”) and DCL2 (“holds out their arms looking for the person they wish to contact”) were eliminated due to their ambiguity and due to their value close to 0 in DI and CHI. In addition, these decisions are also supported by the increase that would occur in Cronbach’s Alpha (*α* = 0.978) when eliminating these elements (*α* > 0.979). The Alpha thus amounts to *α* = 0.98.

### 3.2. Exploratory Factor Analysis

After refining the items by removing LCS1 and LCS2, MS4 and MS7, DLS2, as well as DCL1 and DCL2 in the previous analysis, an exploratory factor analysis (EFA) was performed using the principal axis factoring method and varimax rotation on the results of the original version of the instrument. The results of Bartlett’s test of sphericity (*χ^2^*_2556_ = 13848.25; *p* > 0.001) and the Kaiser-Meyer-Olkin test (KMO = 0.930) allowed us to rule out that the correlations between items constituted an identity matrix. The value of the determinant of the correlations (D = 1.463) was considered, indicating low intercorrelations between variables. An initial solution was obtained in 10 factors, which explained 64.69% of the total variance. However, given the interest in the search for a more parsimonious solution, based on a reduced number of factors, we found that 50% of the variance accumulated in the first four factors (54.89%).

After analyzing the factorial weight of the items in each factor, items with weights lower than 0.4 were observed, which, therefore, had a low explanatory power of the dimension they are part of, and their elimination is recommended [45]. This was the case of item LCS3 (“When called by name, turns head towards the caller”), DLS2 (“Picks up and eats food such as cookies or potato chips”), DLS3 (“Lengthens arms and legs when dressed, to facilitate the task”), and DLS6 (“Remains without urinating for at least 3 h”). Also, DLS1 (“Swallows soft food”), CLS1 (“Finds toys or objects that are always kept in the same place”) were the only ones that loaded in factors 9 and 10, not obtaining factorial weights that relate them to the rest of the dimensions. Therefore, their elimination was considered pertinent. Note that the justification for those eliminations can be argued because they are questions referring to basic skills typical of the first years of life (0–2 years). Therefore, their consultation is irrelevant for the sample as a whole, as they are assumed to have been acquired in older persons and can generate confusion for families who are assessing the current moment of adaptive behavior of their family members.

Again, considering the principle of parsimony, the factorial structure was verified by forcing the rotation to four factors which explained 58.52% of the total variance. The highest percentage of variance accumulated in the first three factors, with only 2.58% attributable to the fourth factor. Therefore, the factorial structure with three dimensions was verified, which explained 55.31% of the variance (where factor 1 accumulated 41.24%).

The final structure did not fully conform to the underlying model, but interactions were observed between several of the items in the questionnaire, especially in relation to daily life skills and life in the community. The content of those items could be framed within a general dimension of daily life skills since it includes various skills of relevance to the daily functioning of a person. In the same sense, some daily life and community life skills were grouped within linguistic and communication or motor skills, since they seem to be evaluating more the communication and language development of a person, or of their skills to perform motor actions, than the basic skills themselves.

Table 1, Table 2 and Table 3 present the items of the instrument divided into the three dimensions extracted from the EFA, after making the decision to unify the dimensions into three elements more congruent with the results obtained: motor skills (MS), linguistic and communication skills (LCS), and daily life skills (DLS). The total reliability of the scale was *α* = 0.836.

Table 1 shows a total of 18 items that represent the factor (*M* = 3.43, *SD* = 0.37; *α* = 0.908), of which 12 originally belonged to the instrument, and five corresponded to personal life skills and one to the skills of community life. Having taken that into account, in this case, some of those last items present factorial loads in other factors. This indicates its relationship with other items present in other dimensions. Even so, we adopt the solution that, even with a lower load (e.g., MS18), is more coherent with the original proposal of the instrument and better serves the conceptual content.

Table 2 shows a factor composed of 16 items (*M* = 2.96; *SD* = 0.48; *α* = 0.952), of which 12 corresponded to the original structure, while the remaining three corresponded to the community life skills (two of them) and to the motor skills (the third one). Regarding the content of those items, the indication of the completion of a task, the correct use of temporal terms, and correct writing, although linked to motor skills, they also present a clear link with linguistic and communication skills, justifying the relevance of its location in this space.

The daily life skills dimension is the one that contains the highest number of items, with a total of 27 (*M* = 2.96; *SD* = 0.48; *α* = 0.95), which is justified by being a dimension that brings together two dimensions of the original questionnaire, referring to daily life skills and community life skills. Note that some items have loadings on several factors, showing their relationship with items from other dimensions. Item CLS4 stands out as it has higher loadings on F2 than on F1. Even so, we chose to include it in F2 as it is closer to the original proposal of the instrument and has greater conceptual coherence with the dimension.

Finally, and given the high number of items that make up each dimension, we have proceeded to the itemization, as presented in Table 4, using a new EFA with the principal axis factoring method and varimax rotation to group, within each dimension, the items with the greatest conceptual similarity and thus make an initial approach to a future confirmatory validation of the instrument.

## 4. Discussion

The present study aims to evaluate adaptive behavior through the ICAP as well as the behavior of this instrument in terms of validity and reliability in clinical practice with people with ASD, in order to verify compliance with the requirements rigorously required, as well as seek improvements for it, observing overlapping of information in the dimensions considered both in the original instrument, and in its translated and adapted version into Spanish, used in the present study [40].

If we look at the previous research, it is observed that most of the definitions focused on adaptive behavior indicate a relevant factor referred to the development of the personal autonomy skills demanded [7,14] to satisfy the most basic needs (food, personal hygiene, clothing…) and, in the present study, they have been conceptualized within a large dimension called daily life skills which, with excellent reliability (α = 0.892–0.935), brings together both the most basic hygiene, care, and feeding skills as well as the most advanced ones of life in society and autonomy.

A second element highlighted in the literature refers to the skills necessary to be an active member of society (traveling independently, expressing oneself through language, learning precise skills to perform a job, to communicate in a functional way, etc.); along with this, Coulter and Morrow [46] refer to a third element related to the ability to maintain responsible social relationships. Unifying those two elements, we find a second dimension in the present study that shows excellent reliability (α = 0.860–0.931) and is conceptualized as communication and linguistic skills. Although it also links at a certain point, such as in the dimensions of daily life, mainly reflects the person’s ability to use language or symbols for communication, expressive-receptive communication, and for the use of pragmatics. The three areas proposed in most of the proposals of the scientific literature about behavior refer to independent functioning or self-sufficiency, interpersonal relationships, and social responsibility, and in the present work have been clearly collected in those two broad and full dimensions. Regarding the motor skills dimension, which presents good reliability (α = 0.828–0.857), numerous studies reveal its significant relationship with adaptive behavior in children with neurodevelopmental disorders such as ASD [21] and support the distribution in the two subcategories of fine and gross motor skills performed in this work [42].

It should be noted that a fourth area has been the subject of debate and controversy, even radically among some such as Dell’Armo and Tassé [2] in favor of including it in the construct of functional academic skills (reading and calculation, among others) and those who, such as Mercer [47] points out that one of the main uses of adaptive behavior is to evaluate skills other than school ones. The reason given by the latter is that adaptive behavior should be a means to avoid bias in the evaluation derived from the prominence exercised by the intelligence and performance tests observed in the case of children from ethnic minorities or disadvantaged social environments [38]. The differences between adaptive behavior in school and outside of it have been empirically proven [41], even in more recent studies, in which, knowing the difficulty in evaluating adaptive behavior, the focus is on functional, applied, or general skills, including independence [7], flexibility [10], executive functioning [16], among others.

Hence the need to propose studies that, from the theoretical-practical point of view, allow detecting the psychometric validity of the dimensions that make up the construct of adaptive behavior, as well as identifying the difficulties that exist in each of the areas to provide adequate support in each of them avoiding false positives (reducing the possible over-representation of people from minority groups) and false negatives who may not be receiving a relevant and necessary service [48], as well as offering information on possible methodological errors that affect its use in clinical practice [15].

Considering the results obtained from the ICAP analysis, it can be pointed out that it continues to be a widely useful tool for research, but especially for diagnosis and care. However, the need to review and update its contents has become clear, reducing the high number of items (77), and adapting its contents to the current situation. In the case of the present study, an approach is provided to a possible grouping in three large dimensions of adaptive behavior, with three subdimensions each and a total of 60 items, with excellent internal consistency indices comparable to those obtained in studies carried out in recent years with this instrument outside of Spanish territory [35,42].

The evaluation of adaptive behavior implies establishing a diagnosis of intellectual disability, so the use of standardized instruments by professionals should make it possible to detect significant differences in adaptive behavior on the standardized criterion of a differential score of two standard deviations below half. Following Luckasson and colleagues [47], said instruments for this must meet three requirements: (a) that they are psychometrically validated; (b) determine the three areas or domains originally proposed by the AAMR and maintained by the AAIDD in its definition of adaptive behavior based on conceptual, social, and practical skills; and (c) be standardized in groups and people with intellectual and neurotypical disabilities. Through this study, a response has been made to those three requirements formulated here and supported by subsequent researchers, revealing through the analysis carried out, the validity of the instrument to assess adaptive behavior, provided that the relevant changes are made.

Precisely, the contribution of this research is observed in a double sense: on the one hand, in accounting for the interest and usefulness of an instrument that, after decades of research and analysis on adaptive behavior, continues to be useful and valid in our country; and, on the other hand, in trying to offer improvements to it by reducing the high number of items that the original instrument presents and carrying out a search for a more comprehensive definition of adaptive behavior. It is evident that the study is not exempt from limitations, such as the fact of not having achieved a smaller instrument or a second application of it, although it is planned in future research, as well as the confirmation of the factorial structure presented based on the presence of first and second order factors. Likewise, the fact that we have focused our efforts on analyzing the factor structure of the ICAP has hindered the analysis of convergent/divergent validity, in line with previous studies in other Spanish-speaking contexts [35], and this is a point to be followed up in subsequent applications of the instrument to check its correlation with other scales focused on assessing adaptive behavior or some of its dimensions (daily living skills, communication, and language, or motor skills).

In short, the contemporary development of adaptive behavior assessment instruments, such as the adaptation and improvement of the ICAP, as well as research contributes to accurately identifying ASD and other developmental disabilities and understanding adaptive behavior in its diagnosis and care. The process of conceptual and theoretical change must involve an effort to identify and improve the resources and evaluation instruments, and the professional frameworks for the diagnosis and the development of supports.

## Figures and Tables

**Table 1:** Motor skills dimension: items and factor load.

Item *	F1	F2	F3
MS3 Sits alone, keeping their head and back straight and steady for 30 s.			0.591
MS5 Stands up by himself/herself.			0.658
MS6 Puts small objects into containers and removes them later.			0.544
MS8 Makes lines, marks, or scribbles, with pencil or paints, on a sheet of paper.			0.448
MS9 Unwraps small objects, such as gum or candy.			0.533
MS10 Turns the door handles and opens them.			0.655
MS11 Goes up and down the stairs alternating his/her feet from one step to another (even with support).			0.768
MS12 Climbs a ladder two meters high (e.g., of a slide or a step ladder).			0.719
MS13 Cuts with scissors following a thick straight line.		0.412	0.431
MS15 Lifts and carries a bag full of groceries at least twenty feet away and places it on the ground.			0.536
MS16 Folds a letter into three equal parts, puts it in an envelope, and then closes it.		0.516	0.565
MS18 Assembles objects of at least ten pieces, which must be screwed or fitted together with nuts and bolts (e.g., toys and disassembled furniture).	0.495		0.436
DLS4 Keeps hands under running water to wash them when in front of a sink.			0.414
DLS7 Takes off pants or skirt and underwear.		0.435	0.556
DLS8 Evacuates when sitting on the toilet on a regular schedule or when taken to the bathroom.		0.424	0.439
DLS9 Wears t-shirts or jerseys, even if it is the other way around.		0.439	0.459
DLS10 Makes use of the toilet, taking off and putting on clothes.		0.454	0.516
CLS2 Goes alone to a certain room when commanded.			0.427

* Acronyms: F—Factor; MS—Motor Skills; DLS—Daily Life Skills; CLS—Community Life Skills.

**Table 2:** Linguistic and communication skills dimension: items and factor load.

Item *	F1	F2	F3
LCS4 When asked, imitates actions, such as saying goodbye or clapping.		0.595	
LCS5 Gives toys or objects to another person.		0.512	
LCS6 Indicates “yes” or “no” by shaking the head or otherwise, to answer simple questions, such as “do you want milk?”		0.571	
LCS7 Points to objects or people on a picture, when asked.		0.719	
LCS8 Says at least 10 words, which can be understood by someone who knows them well.		0.718	
LCS9 Asks simple questions.		0.843	
LCS10 Speaks using three- or four-word phrases.		0.823	
LCS11 In group activities, wait at least two minutes for their turn to come.		0.574	
LCS12 Offers help to other people (e.g., holds the door).	0.455	0.532	
LCS13 Behaves in an appropriate manner, without attracting the attention of others, when with friends in public places.		0.553	
LCS14 Reacts correctly to most of the most common signs, labels, or symbols.	0.407	0.732	
LCS15 Tells a story in a summarized way so that another person can understand it (e.g., a TV program or a movie).	0.491	0.653	
CLS3. Indicates when a routine task has been completed or was commissioned.		0.667	
CLS5 Correctly uses the words morning and night.		0.760	
CLS10 Tells the complete date of his/her birth (day, month, and year).	0.508	0.570	
MS14 Write his/her name by copying it from a model.		0.676	

* Acronyms: F—Factor; LCS—Linguistic and Communication Skills; CLS—Community Life Skills; MS—Motor Skills.

**Table 3:** Daily life skills dimension: items and factor load.

Item *	F1	F2	F3
DLS5 Eats solid foods using a spoon, without spilling almost anything.	0.401		
DLS13 Uses a knife to cut food, instead of trying to eat pieces that are too large.	0.605	0.482	
DLS14 Soaps up, rinses, and dries hair.	0.582		
DLS15 Scrubs, dries, and then puts the dishes in place.	0.669		
DLS16 Prepares and combines simple meals, such as fried eggs, soup, or sandwiches.	0.723		
DLS17 Tidies up his/her bedroom, including putting away clothes, changing sheets, dusting, and sweeping the floor.	0.714		
DLS18 Prepares shopping lists of at least six items to purchase at a grocery store.	0.819		
DLS19 Loads and operates a washing machine, using the appropriate amount of detergent and program.	0.824		
DLS20 Plans, prepares, and serves a complete meal for more than two people.	0.846		
DLS21 Carries out minor clothing repairs, such as darning a tear or sewing on a button, or, in any case, asks the right person to do it.	0.791		
CLS4 Remains without walking away for ten minutes, in a yard or park without fences, when expected to do so.	0.419	0.464	
CLS6 Exchanges an object for money or for another object of value (e.g., book for another book or money).	0.544	0.477	
CLS7 Buys objects, which cost at least twenty-five pesos/pesetas, in vending machines (e.g., sweets or soft drinks).	0.688		
CLS8 In his/her neighborhood, crosses streets, avenues, or intersections without signposting, without anyone accompanying him/her.	0.737		
CLS9 Buys the things he/she is asked to when on an errand, although he/she may not count the change correctly.	0.692	0.414	
CLS11 Daily uses a clock to do things at a certain time (e.g., take a bus or watch a television program).	0.711		
CLS12 Accurately counts the change, after buying something with a bill/coin of five hundred pesos/pesetas.	0.772		
CLS13 Handles potentially dangerous power tools and appliances (e.g., mixer, drill).	0.759		
CLS14 Complies with appointments that have been set at least three days in advance, writing them down if necessary.	0.659		
CLS15 Manages his/her money in such a way that it covers the expenses of at least one week (leisure, transportation).	0.836		
CLS16 Works on a task at a regular pace, for at least two hours.	0.714		
CLS17 Fills out forms and attends selection interviews to look for work.	0.774		
CLS18 Receives invoices by mail and makes payments before the deadline.	0.789		
CLS19 Makes a monthly balance of his/her bank account or savings book.	0.781		
LCS16 Remembers or knows how to locate phone numbers and calls friends.	0.646	0.485	
LCS17 Writes, by hand or types, legible and understandable notes, or letters, to be sent by mail.	0.663	0.519	
LCS18 Finds the information he/she needs in the yellow pages of the phone book or in the newspaper classifieds.	0.803	0.401	

* Acronyms: F—Factor; DLS—Daily Life Skills; CLS—Community Life Skills; LCS—Linguistic and Communication Skills.

**Table 4:** Final exploration of the sub-dimensions of the questionnaire: items and reliability by subscale.

Dimension	Indicator	Total Variance Explained	No. Items	Items	α
Motor skills	Initial gross and fine motor skills	37.91	7	MS3, MS5, MS6, MS8, MS10, MS11, MS12	0.847
	Intermediate gross and fine motor skills	9.66	6	DLS4, DLS7, DLS8, DLS9, DLS10, CLS2	0.857
	Advanced gross and fine motor skills	4.73	5	MS9, MS13, MS15, MS16, MS18	0.828
Linguistic and communication skills	Use of language/symbols for expressive communication	55.01	7	LCS9, LCS10, LCS14, LCS15 MS14, CLS5, CLS10	0.931
	Use of language/symbols for receptive communication	6.27	4	LCS4, LCS5, LCS6, LCS7	0.860
	Pragmatics of language	4.44	4	LCS11, LCS12, LCS13, CLS3	0.844
Daily life skills	Basic life skills	57.7	11	LCS16, LCS17, CLS4, CLS6, CLS7, CLS8, CLS9, CLS11, CLS12, CLS14, CLS16	0.892
	Advanced skills for autonomous living	5.86	8	LCS18, DLS5, DLS20, CLS13, CLS15, CLS17, CLS18, CLS19	0.922
	Basic grooming, feeding, and care skills	4.00	8	DLS13, DLS14, DLS15, DLS16, DLS17, DLS18, DLS19, DLS21	0.935

Acronyms: DLS—Daily Life Skills; CLS—Community Life Skills; LCS—Linguistic and Communication Skills; MS—Motor Skills.

## Data Availability

Not applicable.
